# Improved strategy for large scale isolation of sialylglycopeptide (SGP) from egg yolk powder

**DOI:** 10.1016/j.mex.2019.04.007

**Published:** 2019-04-09

**Authors:** Kathirvel Alagesan, Daniel Kolarich

**Affiliations:** a Department of Biomolecular Sciences, Max Planck Institute of Colloids and Interfaces, 14476 Potsdam, Germany; b Institute of Chemistry and Biochemistry, Freie Universität Berlin, 14195 Berlin, Germany; c Institute for Glycomics, Griffith University, Gold Coast Campus, QLD, 4222, Australia; d ARC Centre for Nanoscale BioPhotonics, Australia

**Keywords:** SGP, sialylglycopeptide, DNA, deoxyribonucleic acid, MS, mass spectrometry, MS/MS, tandem mass spectrometry, ESI, electrospray ionization, Improved strategy for large scale isolation and purification of sialylglycopeptide from egg yolk powder, Synthetic glycobiology, Glycopeptide isolation, Egg yolk powder

## Abstract

Chicken egg yolk is an easily available source for the isolation of sialylglycopeptides (SGP) carrying homogenous biantennary *N*-glycans. This approach has gained much attention in the last decade since these SGPs can easily be used for the semi-synthesis of glycoconjugates circumventing laborious full-synthetic methodologies. Here we report an optimised, significantly shorter (one day instead of five) and environmentally friendly procedure for the mg scale isolation of SGP using commercially available egg yolk powder. A single chromatographic step following chloroform/methanol precipitation of proteins and lipids yielded desired approximately 200 mg SGP from 250 g egg yolk powder within a day.

• Environmentally friendly procedure for isolation of sialylglycopeptide from Egg yolk powder.
• Reduced the protocol from five days down to one.

## Graphical abstract

**Figure:**
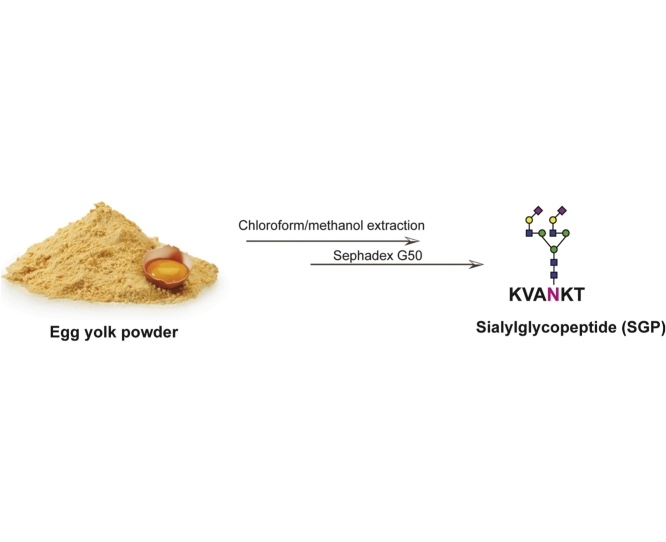

**Specifications Table**

**Table:** 

Subject Area:	***Agricultural and Biological Sciences***
More specific subject area:	Natural product isolation Analytical glycobiology
Method name:	Improved strategy for large scale isolation and purification of sialylglycopeptide from egg yolk powder
Name and reference of original method:	This method was significantly modified for isolating the glycosylated hexapeptides as described by Seko et al. ‘***Occurence of a sialylglycopeptide and free sialylglycans in hen's egg yolk*’**. Biochim Biophys Acta. 1997 Apr 17;1335(1-2):23-32. The herein described improved method uses the chloroform/methanol method described by Wessel, D. and U.I. Flugge, ‘***A Method for the Quantitative Recovery of Protein in Dilute-Solution in the Presence of Detergents and Lipids’***. Analytical Biochemistry, 1984. 138(1): p. 141-143., as a replacement for the phenol extraction used by Seko et al. for isolation of the desired glycosylated hexapeptide from egg yolk powder.
Resource availability:	N/A

## Method details

The ability to produce defined and well-characterised compounds by synthetic approaches has always been a major driving force in science. The establishment of synthetic routes allowing the production of defined DNA or peptide sequences in high yields and purity has been crucial for the development of biosciences and our current understanding of cellular functions [1,2]. However, to date the synthetic capacities to easily produce a large variety and quantity of synthetic glycopeptides is still lagging behind classical peptide or DNA synthesis strategies, also because of the large structure complexity associated with glycans.

Despite tremendous advances in carbohydrate chemistry [[3], [4], [5]] the production of large oligosaccharides with diverse building blocks still requires substantial time and material resources. In particular, introduction of certain biologically important glyco-features such as α-fucose, α-sialyl or β-mannose linkages between the monosaccharide building blocks are challenging and represent major limiting steps in the production of sufficient quantities of larger oligosaccharides such as *N*-glycans. Despite these challenges, total complete synthesis of glycopeptide bearing fucosylated biantennary, disialylated *N*-glycans was in principle accomplished by the Danishefsky research group [6], though with considerable efforts that yet make it unfeasible for routine production.

One way of circumventing these obstacles is to make use of nature's glycosylation potential by isolating the compounds of interest from natural resources such as egg yolk [[7], [8], [9], [10], [11]]. These compounds can easily be purified and subsequently transformed into protected building blocks, which then can be used for glycoconjugate synthesis [[7], [8], [9], [10], [11]]. This approach is significantly more cost effective, quicker and allows the production of g-quantities within just 2–3 weeks. Thus, having a methodology in hand that allows easy production of the necessary precursor for chemo-enzymatic synthesis of glycoconjugates will significantly contribute towards better understanding of the biological role of glycoconjugates and their analysis [[12], [13], [14], [15], [16]]. Here we present the significant improvement from earlier strategies [7,[17], [18], [19], [20]] that reduces the time to isolate milligram quantities of glycosylated amino acid precursors from egg yolk by 80%. The here described simple and environmentally friendly isolation strategy provides the crucial precursor building blocks necessary for step-wise chemo-enzymatic synthesis glycoconjugates required for functional and analytical glycobiology.

## Materials

• Egg Yolk Powder was obtained from Myprotein online shop (Greater Manchester, England - http://www.myprotein.com/home.dept)
• Methanol (Sigma-Aldrich) Caution: Methanol is toxic and highly flammable, and it should be handled in the fume hood. Ensure proper functionality of the fume hood and that there are no open flames or spark-generating devices nearby while handling this chemical. The minimum Personal Protective Equipment (PPE) for work with Methanol is Nitrile laboratory gloves, lab coat, and safety glasses.
• Chloroform (Sigma-Aldrich) Caution: Chloroform is harmful and should be handled in the fume hood. Ensure proper functionality of the fume hood. The minimum PPE for work with chloroform is Viton or PVA (Polyvinyl Acetate) laboratory gloves, lab coat, and safety glasses.
• MilliQ Water
• Centrifuge at 4 °C (Eppendorf 5804-R)
• 50 ml falcon tubes
• RotoVap
• Sephadex G50 (fine) (GE Healthcare Life Sciences)
• Ammonium acetate (Sigma-Aldrich)

## Procedure

Seko and co-workers first reported the isolation of a SGP from fresh chicken egg yolks in milligram scale quantities [17]. However, this approach came with the major drawback that large amounts of phenol were required representing significant downstream processing issues as phenol waste represents a considerable environmental hazard. Here we propose an alternative strategy to isolate a SGP and also show that similar yields could be achieved from commercially available chicken egg yolk powder using a chloroform/methanol protocol [21] that concomitantly precipitated proteins and removed lipids into different fractions and also allowed recycling of the used organic solvents.

## Sialylglycopeptide extraction

1) Resuspend 250 g of egg yolk powder in 750 ml of MilliQ (MQ) water (1: 3 = W/V ratio) and stir the suspension for 2 h at room temperature.

Note: resuspension step can also be carried out overnight if required at room temperature

1) Add, 500 ml of Methanol (2 vol) to this mixture and stir for another 1 h at room temperature.
2) Separate the suspension into fractions of 40 ml using 50 ml falcon tubes.
3) Centrifuge at 3000 x g at 4 °C for 5 min
4) Add 10 ml chloroform to the egg yolk-Methanol mixture and mix well
5) Centrifuge at 3000 x g at 4 °C for 10 min
6) Carefully, decant the aqueous phase containing the desired product and concentrate the pooled fractions under reduced pressure using a rotovap.

## Purification

The obtained crude product was volume reduced using rotovap and the concentrated solution was subjected to gel filtration on Sephadex G50 (fine) (25 X 935 mm) and eluted with 100 mM Ammonium acetate (pH 7) at the flow rate of 1 ml/min at 10 °C. Sixty fractions of 10 ml were collected, and the resorcinol positive fractions pooled.

## Method validation

**Fig. 1:**
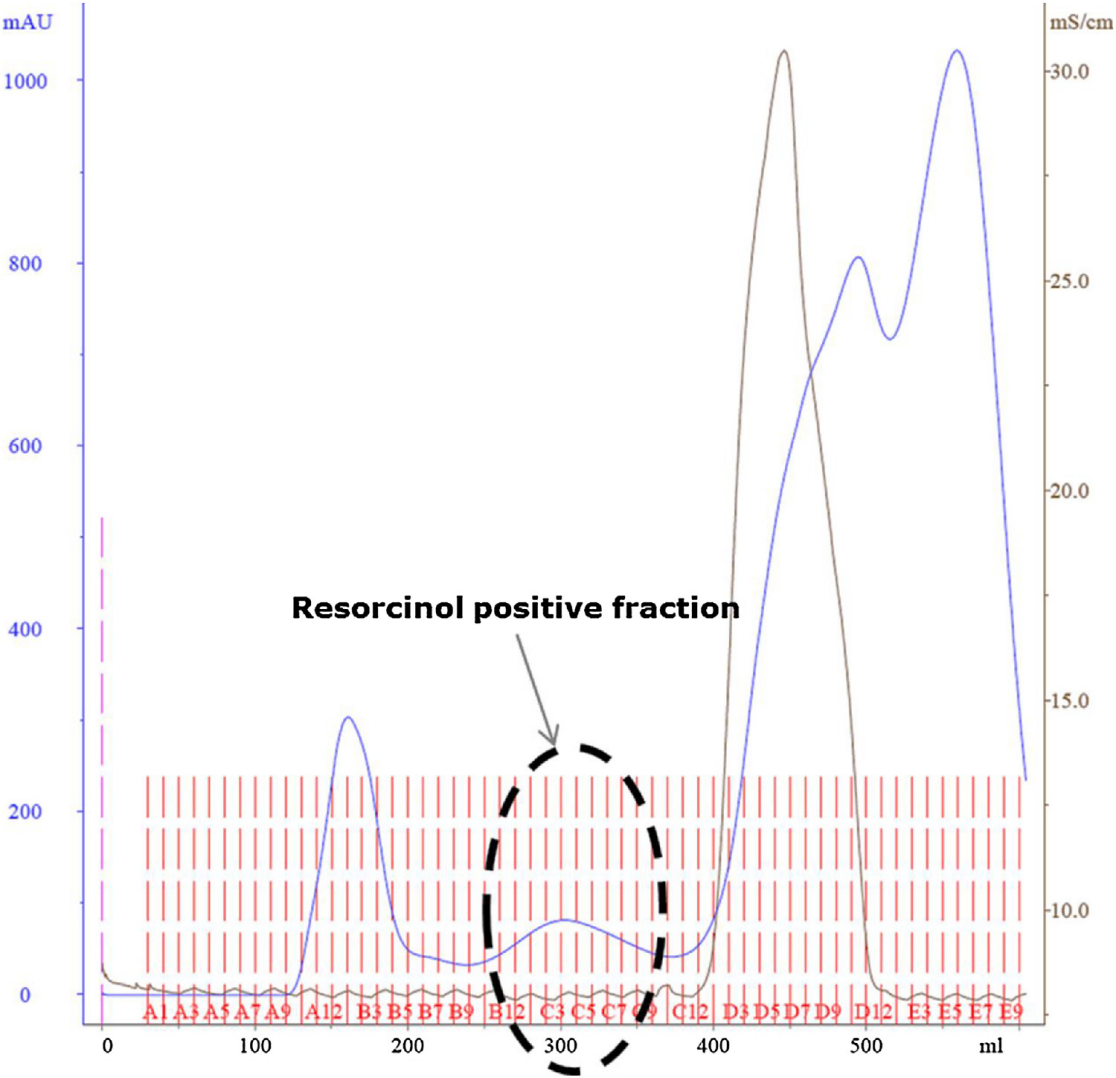
Chromatogram showing the separation of the glycosylated hexapeptide after Sephadex G-50 (fine) gel chromatography (Flow rate: 1 ml/min, UV: 280 nm (blue solid line) and solvent: 100 mM ammonium acetate, red dashed line indicates the fraction number, brown solid line indicates the conductivity), which can be clearly separated from different larger and smaller components (peaks before and after the resorcinol positive fractions).

**Fig. 2:**
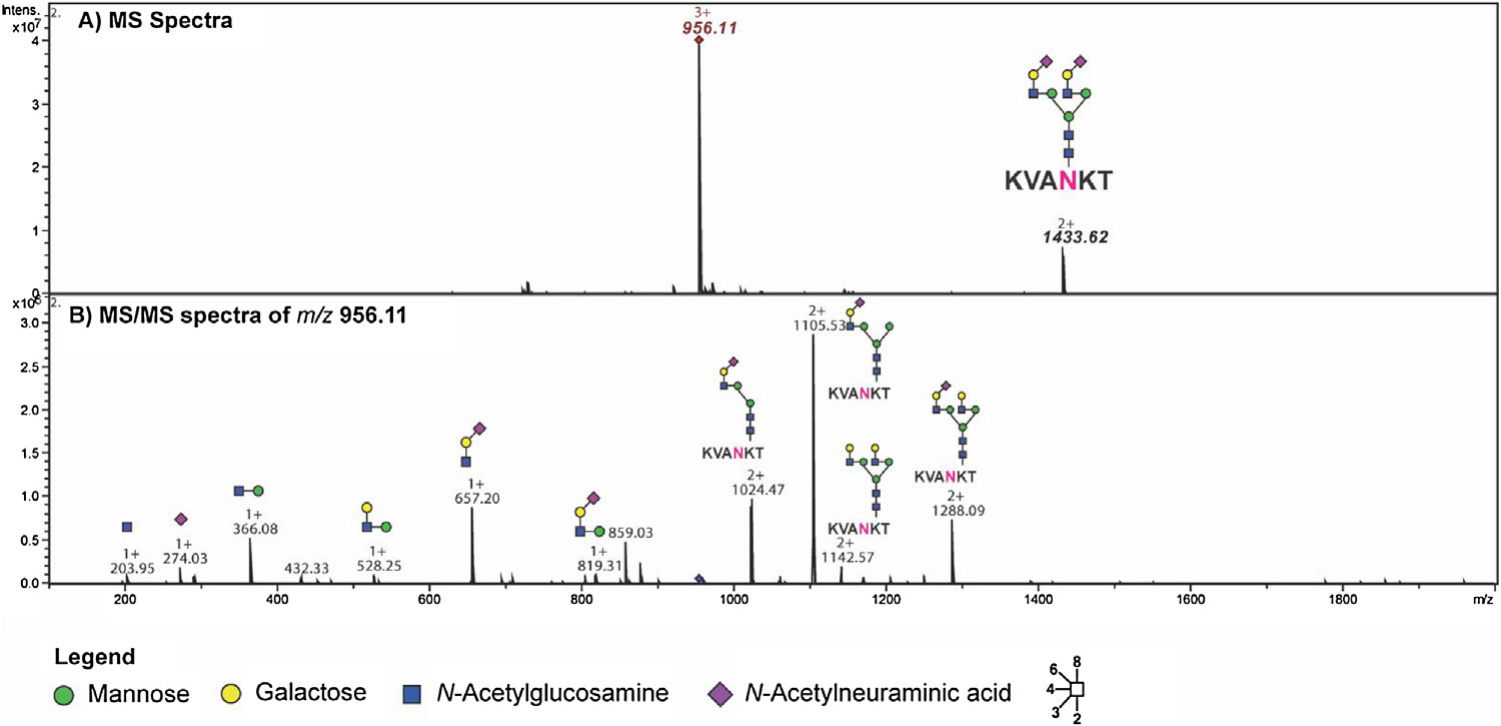
MS and MS/MS spectra of SGP isolated from egg yolk powder. An aliquot (10 μl) of resorcinol positive fractions was pooled after gel filtration. This sample was diluted in 1000 μl of 30% aq.ACN containing 0.1% formic acid and analysed via offline MS using an amaZon ion trap mass spectrometer at the flowrate of 1 μl/min (MS and MS/Ms scan range 100–2000). (A) Summed MS spectra indicate the high purity and presence of a homogenous SGP isolated from egg yolk powder. The signals at *m*/*z* 956.11 [M+3H]^3+^ and *m*/*z* 1433.62 [M+2H]^2+^ correspond to the egg yolk hexapeptide carrying a sialylated biantennary N-glycan further verified by tandem MS spectra of *m*/*z* 956.11 (bottom spectrum).

This novel isolation workflow comes with an additional advantage that just a single chromatographic step after chloroform/methanol precipitation is sufficient to yield the glycosylated hexapeptide and the isolated product was verified by ESI-MS/MS (Fig. 1, Fig. 2). The ratio used for extraction of the glycosylated hexapeptide from egg yolk enabled a quick and easy isolation of approx. 200 mg glycopeptide from 250 egg yolk (Table 1). One of the biggest advantages of the procedure is that the chloroform layer containing the lipids can easily be reused after a simple distillation, thus tremendously reducing the amounts of organic solvents required for glycopeptide purification and reducing organic waste in general.

**Table 1:** Comparison of various methods available for SGP isolation from either fresh egg yolk or egg yolk powder.

Parameters	Seko A - 1997	Zou Y- 2012	B Sun - 2014	L Liu - 2017	DKA approach
**Starting material**	Fresh 30 egg yolks	100 egg yolk	300 egg yolk	2.27 kg egg yolk powder	250 g dried egg yolk powder
**No of Steps**	**7**	**3**	**7**	**5**	**2**
**Duration for extracting hexapeptide**	5 days	Information not provided by authors	3-4 days^a^	Information not provided by authors	1 day
**Solvent used**	Phenol	Phenol	Ethyl ether, acetone	Ethanol	Chloroform, methanol
**Yield**	300 mg	680 mg	1.9 g	1.82 g	200 mg
	**8 mg/egg yolk**	**6.8 mg SGP/egg**	**0.9 mg SGP/egg yolk powder**	**0.8 mg SGP/g of egg yolk powder**	**0.8 mg SGP/g of egg yolk powder**

a Based upon the information available in Tang F et al. [22].
